# Nutritional status and its determinants in the Eastern Mediterranean region. *A review*

**DOI:** 10.15537/smj.2022.43.4.20210676

**Published:** 2022-04

**Authors:** Emad A. Al-Shameri, Ahmed H. Al-Shahethi, Sharifah W. Wafa

**Affiliations:** *From the School of Nutrition and Dietetics (Al-Shameri, Wafa), Faculty of Health Sciences, Universiti Sultan Zainal Abidin, Terengganu; and from the Department of Social and Preventive Medicine (Al-Shahethi), Faculty of Medicine, University of Malaya, Kuala Lumpur, Malaysia.*

**Keywords:** malnutrition, Eastern Mediterranean region, determinants, overweight, stunting

## Abstract

**Objectives:**

To review relevant literature on the determinants of the nutritional status of the children below 5 years of age in the Eastern Mediterranean Regional (EMR) countries and investigate the updates related to risk factors associated with malnutrition.

**Methods:**

A search of pertinent literature and databases was conducted using the PubMed and Google Scholar databases by applying some keywords.

**Results:**

From the available literature reviewed, the mean prevalence of underweight, wasting, and stunting of the children below 5 years of age in the EMR were 12.8% (6.4%-22.6%), 7.5% (5.9%-9.4%), and 24.2% (15.6%-35.5%) respectively. The EMR countries like Sudan, Yemen, Libya, Afghanistan and Pakistan showed the highest rate of stunting amongst the children (>30%). Furthermore, on average, 5.7% of the children were seen to be overweight. Countries such as Lebanon, Egypt, Syria, Libya, and Tunisia reported the maximal prevalence of overweight children. The study also identified a positive and negative relationship between demographic and socioeconomic and health determinants with nutritional status of these children under 5 years old.

**Conclusion:**

In this review, the researchers have highlighted the prevalence of malnutrition in EMR countries. Thereafter, the review findings recommend for prioritization of different policies which aimed to improve the nutritional status of the people.

Malnutrition constitutes a serious health issue in childhood worldwideleading to serious health problems in children under 5 years of age in the developing countries leading to short term effects (morbidity and mortality) and long-term ones (deficient developmental potential and poor intellectual abilities.^1^

However, there has still been a gap of knowledge to identify the prevalence of undernutrition status and determinants factors in the Middle East countries. Hence, this review outlined the prevalence and determining factors that cause health issues like wasting, stunting, overweight, and underweight in children below 5 years of age in the Middle East and Eastern Mediterranean Regions (EMR).

## Methods

In this study, the retrospective design was used. The researchers reviewed the data regarding 4 major nutrition indicators specified by World Health Organizaiton (WHO), such as: i) stunting (height-for-age, z score <-2) ; ii) wasting (weight-for-height, z score <-2); iii) underweight (weight-for-age, z score <-2);^2^ and iv) Prevalence of wasting, stunting, underweight and overweight in the children below 5 years. The prevalence is expressed as (number of existing cases of an event, illness, or disease [at a point in time] divided by total population and is often expressed as a %).

All this data was collected from the different databases such as United Nations International Children’s Emergency Fund (UNICEF) Multiple Indicator Cluster Survey, and the WHO Global Database on Child Growth and Malnutrition. Additionally, many governmental websites like the Ministries of Health and the National Public Health Institutes were reviewed.

The time frame that was selected between January 2019 and January 2020. The countries of the EMR were determined according to the regional classification of the World Bank. The countries in the EMR were categorized based on the income groups in the following manner: high-income countries (HIC), middle-income countries (MIC), and low-income countries (LIC). The keywords were used and search terms relevant to EMR (Afghanistan OR Bahrain OR Djibouti OR Egypt OR Iran OR Iraq OR Jordan OR Kuwait OR Lebanon OR Libya OR Morocco OR Pakistan OR Oman OR Palestine OR Qatar OR Saudi OR Somalia OR Sudan OR Syrian OR Tunisia OR Emirates OR Yemen OR EMR). Malnutrition indicators (stunting OR wasting OR underweight OR overweight). This study used the extraction of the latest indicators for the various study variables, from the WHO, UNICEF, and so on, and no typical criteria for inclusion and exclusion have been pre-defined.

### Statistical analysis

The study data were analyzed using SPSS Statistics for Windows, version 25 (IBM Corp., Armonk, N.Y., USA). Statistical analysis was implemented to find out the correlation between variables such as demographic, health and nutritional status including stunting, underweight, wasting and overweight, using the rank-order procedures.

## Results

There is great variation and disparity in the stunting, underweight, wasting and overweight between and within countries of the region Table 1.

**Table 1:** - Prevalence and occurrence of malnutrition amongst the children below 5 years in the Eastern Mediterranean Regional countries.

**ID**	**Country**	Stunting*	**Underweight^†^**	**Wasting^‡^**	**Overweight^§^**
1	Bahrain (1995)	13.6	7.6	6.6	4.9
2	Lebanon (2004)	16.5	4.2	6.6	16.7
3	Egypt (2014)	22.3	7.0	9.5	15.7
4	Tunisia (2018)	8.4	1.6	2.1	17.2
5	Oman (2017)	11.4	11.2	9.3	4.2
6	Islamic Republic of Iran (2010)	6.8	4.1	4.0	11.8
7	Libya (2014)	38.1	11.7	10.2	29.6
8	Saudi Arabia (2007)	9.3	5.3	10.8	6.1
9	Qatar (1996)	11.6	4.8	2.1	10.4
10	Syrian Arab Republic (2009)	27.9	10.4	11.6	17.9
11	United Arab Emirates (1995)	16.7	14.4	15.2	Data unavailable
12	Morocco (2017/18)	15.1	2.6	2.6	10.9
13	Kuwait (2017)	6.4	3.0	2.5	5.1
14	Jordan (2012)	7.8	3.0	2.4	4.7
15	Yemen (2013)	46.4	39.9	16.4	2.5
16	Afghanistan (2018)	38.2	19.1	5.1	4.1
17	Iraq (2018)	12.6	3.9	3.0	6.6
18	Pakistan (2017/18)	37.6	23.1	7.1	2.5
19	Sudan (2014)	38.2	33.1	16.3	3.0
20	Djibouti (2013)	33.5	29.9	21.5	8.1
21	Somalia (2009)	25.3	22.5	14.3	3.0
22	State of Palestine (2014)	7.4	1.4	1.2	8.2
	EMR	24.2 (15.6-35.5) (2019)	12.8 (6.4-22.6) (2019)	7.5 (5.9-9.4) (2019)	7.7 (6.5-9.0) (2019)

Values are presented as percentages. ^*,†,‡^Bahrain, 2000,2018; ^*,†,‡,§^Lebanon,2006; ^*,†,‡,§^Egypt,2015; ^*,†,‡,§^Tunisia, 2018; ^*,†,‡,§^Oman, 2017; ^*,†,‡^Islamic Republic of Iran, 2014, 2018^‡^; ^*,†,‡,§^Libya,2014; ^*,†,‡,§^Saudi Arabia, 2007; ^a,b,c,d^Qatar, 1996; ^*,†,‡,§^Syrian Arab Republic, 2011; ^*,†,‡^UAE, 2018; ^*,†,‡,§^Morocco, 2017/18; ^*,†,§^Kuwait, 2017, 1999/2009^§^; ^*,†,‡,§^ Jordan, 2012; ^*,†,‡,§^ Yemen,2013; ^*,†,‡,§^Afghanistan, 2018; ^*,†,‡,§^ Iraq, 2018; ^*,†,‡,§^ Pakistan, 2017/18; ^*,†,‡,§^Sudan, 2014; ^*,†,‡,§^ Djibouti, 2013; ^*,†,‡,§^ Somalia, 2009; ^*,†,‡,§^ State of Palestine, 2015

Stunting is ranging from 6.4% in Kuwait to 46.5% in Yemen; underweight is ranging from 1.38% in Palestine to 39.0% in Yemen; wasting is ranging from 1.19% in Palestine to 21.5% in Digibuti; and overweight is ranging from 2.5% in Pakistan to 29.6% in Libya.

**Figure 1:**
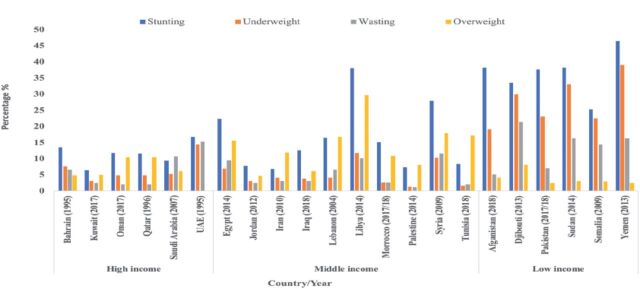
- Prevalence of stunting, wasting, underweight and overweight among children aged <5 years classified by income countries in the Eastern Mediterranean Region between 1995 and 2018.

Figure 1 shows that the highest prevalence of stunting rate was observed in Yemen, Libya, Sudan, Pakistan and Afghanistan, which ranged from 37.6% to 46.5%. The mean annual rate change regarding the prevalence of the stunting issue amongst the children in the EMR area was seen to be -2.8%.

On the other hand, the underweight health issue was more prevalent in Djibouti, Yemen, and Sudan. Data was indicated that the prevalence of wasting in 14 countries is above the acceptable threshold which classifies as per WHO categorisation of the severity of the situation. Some of the EMR countries like Yemen, Syria, Iraq, Libya, and Sudan, who are in the midst of war and political unrest witnessed a higher prevalence of wasting over some time. Additionally, an increased prevalence of the underweight issue was noted in Syrian Arab Republic, Sudan, Yemen, Djibouti, and Libya.

### Health determinant. Table 2

demonstrates that stunting was directly significantly correlated with low birth weight (r=0.57) at *p*<0.01, women anemia (r=0.61) at *p*<0.01, anemia of children <5 years (r=0.66) at *p*<0.01, and inversely correlated with access improved sanitation facility (r= -0.75) at *p*<0.01 while underweight was directly correlated with low birth weight (r=53, *p*<0.05), women anemia (r=0.61, *p*<0.01) and anemia in children >5 years (r=0.73, *p*<0.01) and inversely correlated with access improve sanitation facility (r= -0.58, *p*<0.01).

**Table 2:** - Correlations between nutritional status and demographic-socioeconomic and health determinants.

**Variables/Factors**	**Mean+ SD**	**Stunting**	**Underweight**	**Wasting**	**Overweight**	(***P*** **-value**) **significance**
***Demographic-socioeconomic determinants***						
Population growth (2010-2017) (%)	2.9±2.3	-0.119	0.086	-0.126	-0.455*	<0.05
Total birth (2019) (x1000)	872779.5±1281962.8	0.223	0.199	0.206	-0.344	>0.05
Male literacy rate (2013-2018)	83.7±16.6	-0.687^†^	-0.624^†^	-0.510*	0.358	<0.01; <0.05
Female literacy rate (2013-2018)	73.2±23.2	-0.688^†^	-0.586^†^	-0.488*	0.348	<0.01 <0.05
Total fertility rate/woman	3.3±1.3	0.470*	0.360	0.150	-0.476*	<0.05
Population poverty at national level	29.5±18.1	0.559*	0.509*	0.540*	-0.138	<0.05
***Health determinants***						
Low birth weight (%)	12.9±8.6	0.567^†^	0.534*	0.338	-0.418	<0.05; <0.01
Exclusive breastfeeding (%)	34.3±17.9	0.360	0.344	0.344	0.131	<0.05
Women anaemia (%)	38.2±9.6	0.613^†^	0.611^†^	0.544^†^	-0.549^†^	<0.01
Anaemia of children >5 (%)	36.4±15.7	0.658^†^	0.726^†^	0.552^†^	-0.542*	<0.05; <0.01
Access to improved sanitation facility (%)	80.4±25.9	-0.747^†^	-0.577^†^	-0.534*	0.339	<0.05; <0.01

† Correlation is significant at *p*=0.01 (2-tailed). *Correlation is significant at *p*=0.05 (2-tailed). Total birth (x1000)

In addition, wasting was directly correlated with women anemia (r=0.54, *p*<0.01) and anemia in children <5 years (r=0.55, *p*<0.01) while inversely correlated with access improve sanitation facility (r= -0.53, *p*<0.05).

Lastly, overweight was statistically inversely correlated with women anemia (r= -0.55, *p*<0.01), and anemia of children <5 years (r= -0.54, *p*<0.05). Stunting, underweight, wasting and overweight were not correlated with exclusive breastfeeding.

## Discussion

In this review, we investigated the effect of malnutrition of children below 5 years in the EMR countries, where undernutrition coexisted with overnutrition. Furthermore, it was noted that the regional average (7.5%) was higher than 5%, and was also higher than the values reported in the USA (0.5%).^3,4^ However, the estimates were lower compared to those noted in Asian countries (9.9%), except Japan.^5^ With regards to the underweight health issue, the regional average value (12.3%) was lower compared to those reported earlier in 2005 (16.8%). This indicated that all countries in this region were making attempts to decrease this issue.^6^ The regional average was similar to the global average (13%), whereas it was lower than the value reported in South-East Asian countries (25.5%). Furthermore, stunting was more prevalent in Sudan and Yemen, which indicated that undernutrition was also more prevalent in these countries. The annual prevalence rate of stunting in this region was seen to be -2.8%. This was lower than the stunting rate that was indicated by the 2025 World Health Assembly Global Nutritional Target (-3.9%).^7^

The political conflicts present in the EMR counties has also destabilized the food security network, agricultural production and the livelihood of the people. The countries which are experiencing political unrest have noted an increased prevalence of undernutrition over time.

A higher prevalence of undernutrition in the EMR countries highlights the fact that all the countries need to end different types of food insecurity, increase economic growth and reduce poverty, as highlighted in the Sustainable Development Agenda.^8^

The prevalence of obesity/overweight issues amongst children below 5 years of age has increased in many countries such as Egypt, Iran, Lebanon, Libya, the Syrian Arab Republic and Tunisia. The estimated regional mean value for the overweight issue noted amongst the children <5 years was 5.7%, which was lower than the global average value (5.9%), those reported for Asian countries (5.2%), and the LIC nations (2.7%). However, it was lower than the values reported by the developed countries (7.6%).^9^ This was once regarded as a health issue affecting the HIC nations, however, it has become very prevalent in the LIC and MIC countries, especially in the urban regions.^10^

The main demographic determinants that appeared very strong directly associated with nutritional indicators, the parent’s literacy rate was moderately correlated with the population growth, the total fertility rate and population poverty at the national level. Similar results were reported in an earlier study which indicated that the socio-economic status was related to health issues like wasting, stunting and underweight.^11^

Our results were consistent with a previous study carried out in Rwanda which reported that low birth weight and access to improve sanitation facility associated with stunting among under-5s.^12,13^

The results indicated that an anaemic state in the mother was directly related to the undernutrition status of the child, and led to wasting, stunting, and underweight; while it was inversely related to overweight. Similar results were noted in other study carried out in Nigeria,^14^ who highlighted a significant correlation between the mother’s and child’s hemoglobin levels.

### Study limitations

A possible limitation in this study is the use of secondary data. In addition, the heterogeneity of the countries in the region in that the results could not be applied to all the countries.

These policies can address the malnutrition issue and also help in fulfilling the targets for sustainable development like decreasing poverty, improving economic growth and literacy rates, promoting lifelong learning, developing inclusive societies, ensuring healthy lives and sustainable consumption.^15^

In conclusions, In this review, the researchers characterized the nutritional status of the children below 5 years in EMR countries. They also highlighted the burden of malnutrition affecting these children.
